# Supplementary material for: De Novo Genome Sequence Assembly of the Algal Endosymbiont Micractinium conductrix Derived From Its Host Paramecium bursaria 186b
Source: Genome Biol Evol. 2026 Jul 30;18(9):evag194. doi: 10.1093/gbe/evag194 (PMC13543684; doi:10.1093/gbe/evag194)
Supplement: Supplementary file 1 [file evag194_supplementary_data.zip › 186b_genome_Algal_GBE_SUP_FIGURES1_2.docx]

***De novo* genome sequence assembly of the model algal endosymbiont *Micractinium conductrix* derived from its host *Paramecium bursaria* 186b**

Guy Leonard^1^, Irma Vitonytė^2^, Fiona R. Savory^1^, Erika M. Hansson^2^, Duncan D. Cameron^3^, Michael A. Brockhurst^2^*, Thomas A. Richards^1^*

1. Department of Biology, University of Oxford, Oxford OX1 3SZ, UK
2. Division of Evolution, Infection and Genomics, University of Manchester, Manchester M13 9PT, UK
3. Department of Earth and Environmental Sciences and Manchester Institute of Biotechnology, University of Manchester, Manchester M1 7DN

* Corresponding Authors

**Supplementary Figures 1-2**

**_­­_****_­­_**

**Supplementary Figure 1.** A Blobplot showing the distribution of contigs based on their GC content and coverage (from Illumina reads), blob sizes are indicative of contig size. Colours are assigned from blast taxonomy. It shows three distinct groups of blobs, the larger blobs around 0.65% GC are indicative of the nuclear genome, whereas the two other blobs are suggestive of the mitochondrial and chloroplast genomes.

**Supplementary Figure 2.** A GAQET plot showing various metrics for each of the nine taxa, including the *M. conductrix* 186b RNA-seq informed gene prediction set. Taxa are represented by colours in the key, with radial bar charts per analysis e.g., BUSCO completion, OMARK rank and homology hit rate to Swissprot/TREMBL.
